# Characterization of silver–kaolinite (AgK): an adsorbent for long-lived ^129^I species

**DOI:** 10.1186/s40064-016-1855-8

**Published:** 2016-02-23

**Authors:** Sivachidambaram Sadasivam, Sudhakar M. Rao

**Affiliations:** Geoenvironmental Research Centre, Cardiff University, Cardiff, CF24 3AA UK; Department of Civil Engineering and Centre for Sustainable Technologies, Indian Institute of Science, Bangalore, 560012 India

**Keywords:** Kaolinte, Silver, Thermal reaction, XPS, Radioactive waste

## Abstract

Bentonite is a preferred buffer and backfill material for deep geological disposal of high-level nuclear waste (HLW). Bentonite does not retain anions by virtue of its negatively charged basal surface. Imparting anion retention ability to bentonite is important to enable the expansive clay to retain long-lived ^129^I (iodine-129; half-life = 16 million years) species that may escape from the HLW geological repository. Silver–kaolinite (AgK) material is prepared as an additive to improve the iodide retention capacity of bentonite. The AgK is prepared by heating kaolinite–silver nitrate mix at 400 °C to study the kaolinite influence on the transition metal ion when reacting at its dehydroxylation temperature. Thermo gravimetric-Evolved Gas Detection analysis, X-ray diffraction analysis, X-ray photo electron spectroscopy and electron probe micro analysis indicated that silver occurs as AgO/Ag_2_O surface coating on thermally reacting kaolinite with silver nitrate at 400 °C.

## Background

Bentonite is identified as potential buffer material in deep geological repositories for disposal of high level radioactive wastes (HLW) owing to its very low hydraulic conductivity, large swelling ability and high adsorptive capacity to retain cations (Pusch 2008). However, owing to negative surface charge, bentonite repels anions (van Olphen 1963). ^129^I (Iodide-129) is a fission product encountered in nuclear power plant wastes and is generated from ion-exchange resins, filter sludge, evaporator bottoms, off-gas cartridge filter, trash, and decommissioning wastes (Zhang et al. 2002). The ability to retain iodide ions by bentonite buffer employed in deep geological repositories is important as high-level radioactive wastes contain long-lived radioactive iodide species (Oscarson et al. 1986; Krumhansl et al. 2002).

Treating bentonites with long-chain cationic polymers (example, hexadecyl pyridinium ions, HDPy^+^ ions) have been observed to improve the iodide adsorption capacity of bentonite (Bors 1990; Bors et al. 1994, 1997; Riebe et al. 2005; Kaufhold et al. 2007); however mixing HDPy^+^B (HDPy^+^ treated bentonite) with bentonite was observed to considerably reduce the swell potential of the mix (Rao and Sivachidambaram 2012). A large swelling ability of bentonite is essential to close the pathways for contaminant transport that may develop during placement of the buffer in the deep geological repository (gaps between compacted bentonite block and wall of host rock) or from drying of the clay owing to heat emanating from the HLW canister (shrinkage fissures or cracks). So there is a need to develop a new additive material to bentonite which retains iodide without compromising the physico-chemical properties of bentonite. Though silver ions having affinity with iodide, it may reduce the cation exchange capacity of bentonite when its directly loaded on bentonite. So preparing an mineral composite loaded with silver would improve the iodide retention capacity of bentonite without compromising its physico-chemical properties. Kaolinite is frequently used as host material for the formation of clay-based composites (Patakfalvi and Dékány 2004; Okada et al. 2002). When kaolinite is heated in presence of alkali metal salts, alkali ions are incorporated into the clay structure during the course of dehydroxylation (Kallai 1978). On heating at temperatures below 600 °C, kaolinite reacts with alkali metal salts (MX) according to the equation:

1 $$2{\text{SiO}}_{2} \cdot {\text{Al}}_{2} {\text{O}}_{3} \cdot 2{\text{H}}_{2} {\text{O}} + 2{\text{nMX}} \to 2{\text{SiO}}_{2} \cdot {\text{Al}}_{2} {\text{O}}_{3} \cdot 2{\text{nM}}_{2} {\text{O}} + 2{\text{nHX}} + \left( {2 - {\text{n}}} \right){\text{H}}_{2} {\text{O}}$$

It appears that on set of dehydroxylation (Temperature range from 400 to 550 °C; above dehydration but below dehydroxylation) the clay becomes reactive and concurrently, the liberated water dissolves adjacent salt particles and catalyses the reaction (Kallai 1978). This property of kaolinite could be exploited to incorporate silver compounds on the particle surface as they (example silver oxide) have strong affinity for formation of insoluble halides (Cotton et al. 1995). So in the present work, the silver treated kaolinite material has been prepared as an additive to bentonite to improve iodide retention capacity.

Patakfalvi and Dékány (2004) reported intercalation of silver ions by disaggregating the lamellae of kaolinite using dimethyl sulfoxide (DMSO). Daniels and Rao (1983) observed that 35 meq/100 g, 63 meq/100 g, 83 meq/100 g and 106 meq/100 g of silver ions are sorbed by metakaolinite at temperatures of 25, 255, 275 and 290 °C respectively. Guided by the increased amounts of silver retention by kaolinite at elevated temperatures, the present study focuses on the kaolinite’s influence on the transition element and also discuss the possible reaction mechanism of silver nitrate and kaolinite at 400 °C (Dehydration of kaolinite starts around 400 °C. Kaolinite undergoes complete dehydroxylation at the temperature range of 450–600 °C).

## Methods

Kaolinite supplied by Alminrock, Bangalore, was used in the study. Chemical dissolution of kaolinite specimen revealed that it contains, 51 % SiO_2_, 33 % Al_2_O_3_, 1.0 % Fe_2_O_3_, 0.15 % CaO, 0.13 % MgO, 0.22 % Na_2_O and 0.2 % K_2_O. The kaolinite specimen experienced 13.5 % weight loss on ignition. The clay has cation exchange capacity (CEC) of 2.1 meq/100 g. Analytical reagent grade silver nitrate (AgNO_3_; molecular weight = 169.87 g/mol) was used to prepare the silver–kaolinite specimen.

### Preparation and characterization of AgK specimen

A 20 % silver nitrate–80 % kaolinite mix (on oven-dry mass basis) was heated at 400 °C for 30 min in open crucibles in a temperature controlled furnace (±10 °C). The heating duration was restricted to 30 min as initial kinetic experiments conducted with 20 % silver nitrate–80 % kaolinite mixes illustrated that the amount of silver retained by kaolinite attained equilibrium during this heating period; further, the 20 % silver nitrate–80 % kaolinite mixture (on dry mass basis) was selected for preparation of the silver–kaolinite phase, as experiments with 1, 5, 10, 20, 25 and 30 % silver nitrate–kaolinite mixes (heating temperature and duration = 400 °C and 30 min respectively) revealed that silver retention by kaolinite reaches near equilibrium at this silver nitrate concentration. The further increases of silver nitrate concentration above 20 % yielded less increase in silver uptake by kaolinite (Table 1).

**Table 1:** Silver retention capacity of kaolinite

% of silver nitrate in silver nitrate–kaolinite mix	mg of silver retained/g of kaolinite
1	6.1
5	31
10	69
20	150
25	172
30	175

After desired heating of the 20 % silver nitrate–80 % kaolinite mix, the specimen was repeatedly washed with deionised water, until it is free of unreacted silver. Thermo gravimetric analysis (TGA) and differential scanning calorimetric (DSC) analysis were performed using NETZSCH STA 409 thermal analyzer with a heating rate of 10 °C/min. Thermo gravimetric-evolved gas detection (TG-EGD) analysis was performed on 80 % kaolinite + 20 % AgNO_3_ mix and kaolinite specimens using a Metler-Teledo thermal analyzer model TGA/SDTA851e with Balzers ThermoStar Mass Spectrometer. The TG-EGD analysis was performed to determine the weight loss and the gases evolved during the silver nitrate–kaolinite reaction. The X-ray diffraction patterns of the materials were obtained using Cu Kα line (λ = 0.154 nm) in a Phillips Xpert diffractometer. The chemical state of silver in the AgK specimen was examined using ESCA Thermo Fischer Scientific Multi lab 2000 X-ray photoelectron spectrometer with a monochromatic Al Kα (1486.6 eV) X-ray source. JEOL JXA-8530F Electron Probe Micro analyzer (EPMA) was used to obtain the Wavelength Dispersion Spectrometry (WDS) map of silver retained in AgK specimen.

## Results and discussion

### Thermal analysis

Table 2 presents the weight loss experienced by kaolinite, silver nitrate and kaolinite–silver nitrate mixes on heating at 400 °C for 30 min in an electric furnace. The 80 % kaolinite + 20 % silver nitrate mix experiences 0.92 g weight loss (mass of mix = 10 g comprising of 8 g kaolinite and 2 g of silver nitrate). Upon heating individually, 8 g kaolinite and 2 g silver nitrate experience weight losses of 0.18 and 0.07 g respectively. The much larger weight loss experienced by the kaolinite–silver nitrate mix was attributed to salt-catalysed dehydroxylation phenomena (Kallai 1978). This behaviour was further investigated using thermo gravimetric coupled evolved gas detection (TG-EGD) technique.

**Table 2:** Weight loss measurements of kaolinite, silver nitrate and silver nitrate–kaolinite mixes at 400 °C for 30 min

Material	Initial weight (g)	Weight after heating at 400 °C for 30 min (g)	Weight loss (g)
0.1 g AgNO_3_ + 9.9 g kaolinite (1 % AgNO_3_)	10.04	9.95	0.09
1 g AgNO_3_ + 9 g kaolinite (10 % AgNO_3_)	10.15	9.71	0.44
2 g AgNO_3_ + 8 g kaolinite (20 % AgNO_3_)	10.02	9.1	0.92
2.5 g AgNO_3_ + 7.5 g kaolinite (25 % AgNO_3_)	10.06	9.00	1.06
2 g AgNO_3_	2.01	1.94	0.07
8 g kaolinite	8.01	7.83	0.18

**Fig. 1:**
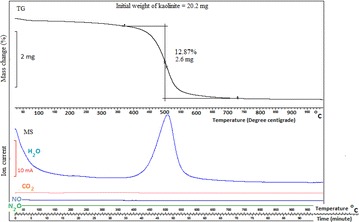
Thermo-gravimetric (TG) along with evolved gas analysis pattern (MS—mass spectrometry ion intensity curve) of kaolinite

**Fig. 2:**
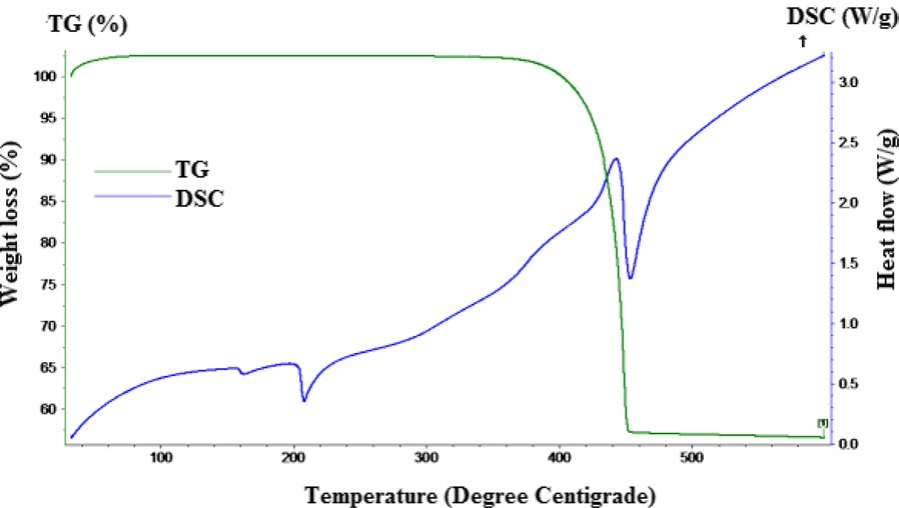
TG and DSC pattern of silver nitrate

The thermo gravimetry (TG) and ion current pattern of evolved gases from kaolinite detected by TG coupled with mass spectrometer (MS) presented in Fig. 1 shows the release of water molecules at the temperature range of 400–600 °C. In this temperature region, 20.2 mg of kaolinite loses 2.6 mg of water, representing 12.97 % weight loss. The TG curve (Fig. 2) shows that the silver nitrate experiences 43 % weight loss at 450 °C and undergoes the reaction:

2 $${\text{AgNO}}_{3} \to {\text{Ag}} + {\text{NO}}_{2} + \frac{1}{2}{\text{O}}_{2}$$

As per the reaction 2, the weight loss should be around 36.5 %. The observed excess weight loss could be the possible influence of atmosphere environment (Otto et al. 2014). The endothermic peak at 212 °C in the DSC curve represents the melting point of silver nitrate, while, the endotherm at 450 °C represents formation of silver metal from reduction of silver nitrate according to reaction 2.

**Fig. 3:**
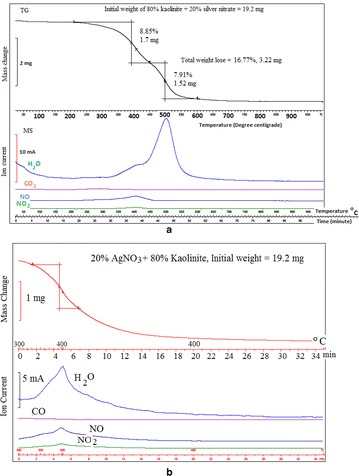
Thermo gravimetric (TG) along with evolved gas analysis pattern (MS—mass spectrometry ion intensity curve) of **a** 80 % kaolinite–20 % silver nitrate mix heated from ambient Temperature to 1000 °C (**b**) 80 % kaolinite–20 % silver nitrate mix heated at 400 °C for 30 min

**Fig. 4:**
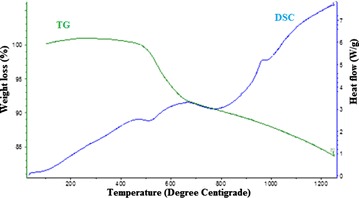
TG and DSC pattern of AgK specimen

The thermo gravimetry (TG) and ion current pattern of 80 % kaolinite + 20 % silver nitrate mix however exhibits a two-step weight loss pattern (Fig. 3a). The first weight loss commences around 212 °C corresponding to melting point of silver nitrate (Fig. 3). The silver nitrate and kaolinite specimens did not show any weight loss around 212 °C (Figs. 1 and 2). In the temperature range of 212–400 °C, the ion current pattern of the kaolinite–silver nitrate mix (Fig. 3) exhibits the release of NO and NO_2_ gases from decomposition of silver nitrate and some release of water molecules from dehydroxylation of kaolinite. This behaviour was further investigated by heating the same composition of the materials at 400 °C for 30 min (Fig. 3b). The Fig. 3b clearly shows the water peak and the NO and NO_2_ gases release at 400 °C and also indicates the reaction rate was well within 30 min time period. In the temperature range of 450–600 °C the second step weight loss occurs (Fig. 3). In this temperature range, ion current pattern shows large release of water molecules from kaolinite. The TG plot also shows that 19.2 mg of 80 % kaolinite + 20 % silver nitrate mix lose 3.2 mg, corresponding to weight loss of 16.76 %. The weight loss observed during step one (8.9 %, Fig. 3) is comparable with the weight loss measurement (9.2 %, Table 2) observed on heating the 80 % kaolinite + 20 % silver nitrate in the furnace at 400 °C for 30 min. The thermo gravimetry (TG) and differential scanning calorimetry (DSC) patterns of AgK specimen in Fig. 4 do not show the endothermic peak at 212 °C (corresponding to melting point of silver nitrate) indicating the absence of free silver nitrate in the AgK specimen.

### X-ray diffraction (XRD) analysis

**Fig. 5:**
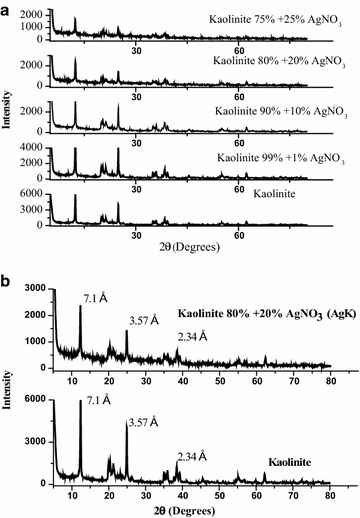
**a**, **b** XRD patterns of kaolinite and AgK specimens

**Fig. 6:**
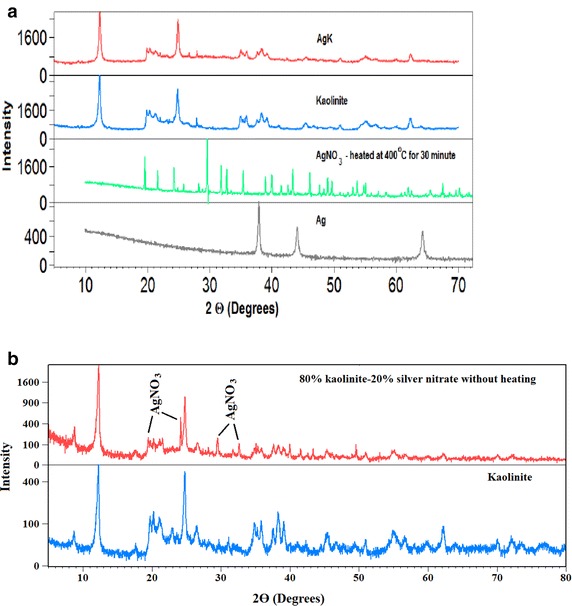
**a** XRD patterns of AgK, Kaolinite, AgNO3 heated at 400 °C and silver (Ag); **b** X-ray patterns of 80 % kaolinite–20 % silver nitrate without heating and kaolinite

**Fig. 7:**
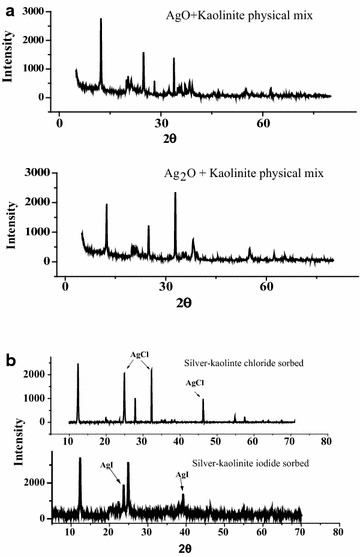
XRD patterns of **a** silver oxides–kaolinite mix and **b** AgK specimens equilibrated with halide ions

XRD patterns of AgK and kaolinite specimens are compared in Fig. 5a, b. The figure shows that the kaolinite mineral in AgK specimen retains its crystalline structure (Fig. 5) as depicted by the strong reflections at 7.1, 3.57 and 2.34 Å (2θ values of 12.45°, 24.91° and 38.4° respectively); comparatively silver nitrate reflections at 2θ values of 19.5°, 24.2°, 29.6° and 32.7° are absent in the XRD pattern of the AgK specimens. The XRD patterns of AgK specimen, silver nitrate heated at 400 °C and silver metal are also compared in Fig. 6a to identify the crystalline form of silver present in AgK specimen. The strong X-ray reflections of silver metal at 2θ values 38° and 44° are absent in the XRD pattern of AgK specimen. The X-ray reflections attribute to silver-nitrate were clearly present in the 20 % AgNO_3_–80 % kaolinite physical mix without heating (Fig. 6a). Further, though silver nitrate heated at 400 °C exhibits reflections at 2θ values 29.6°, 19.5° and 21.67° characteristic of AgNO_3_, these reflections are absent in the XRD pattern of AgK specimen. Similarlly, the XRD patterens of 20 %AgO–80 %kaolinite and 20 %Ag_2_O–80 %kaolinite were exhibits the representative peaks of the silver oxides (Fig. 7a). The absence of crystalline form of silver reflections in the XRD patterns indicating that the silver present in AgK specimen does not occur as Ag metal (or) AgNO_3_ molecule (or) kaolinite influences the X-ray reflections of certain forms of silver compounds (or) the silver oxides retained as amorphous coating on kaolinite surface. The 1 g of AgK specimen equilibrated with 100 mL of 1000 mg/L of chloride and iodide ions exhibited the X-ray reflection peaks attributed to the respective silver halides (Fig. 7b). This behaviour shows that the silver could be retained on kaolinite surface as oxide coating and interacts with halide ions as follow (Cotton et al.^1995^).

3 $$AgNO_{3} + H_{2} O \to AgOH + OH^{ - }$$

4 $$AgOH + I^{ - } \to AgI + OH^{ - }$$

### X-ray photo-electron spectroscopy (XPS) analysis

**Fig. 8:**
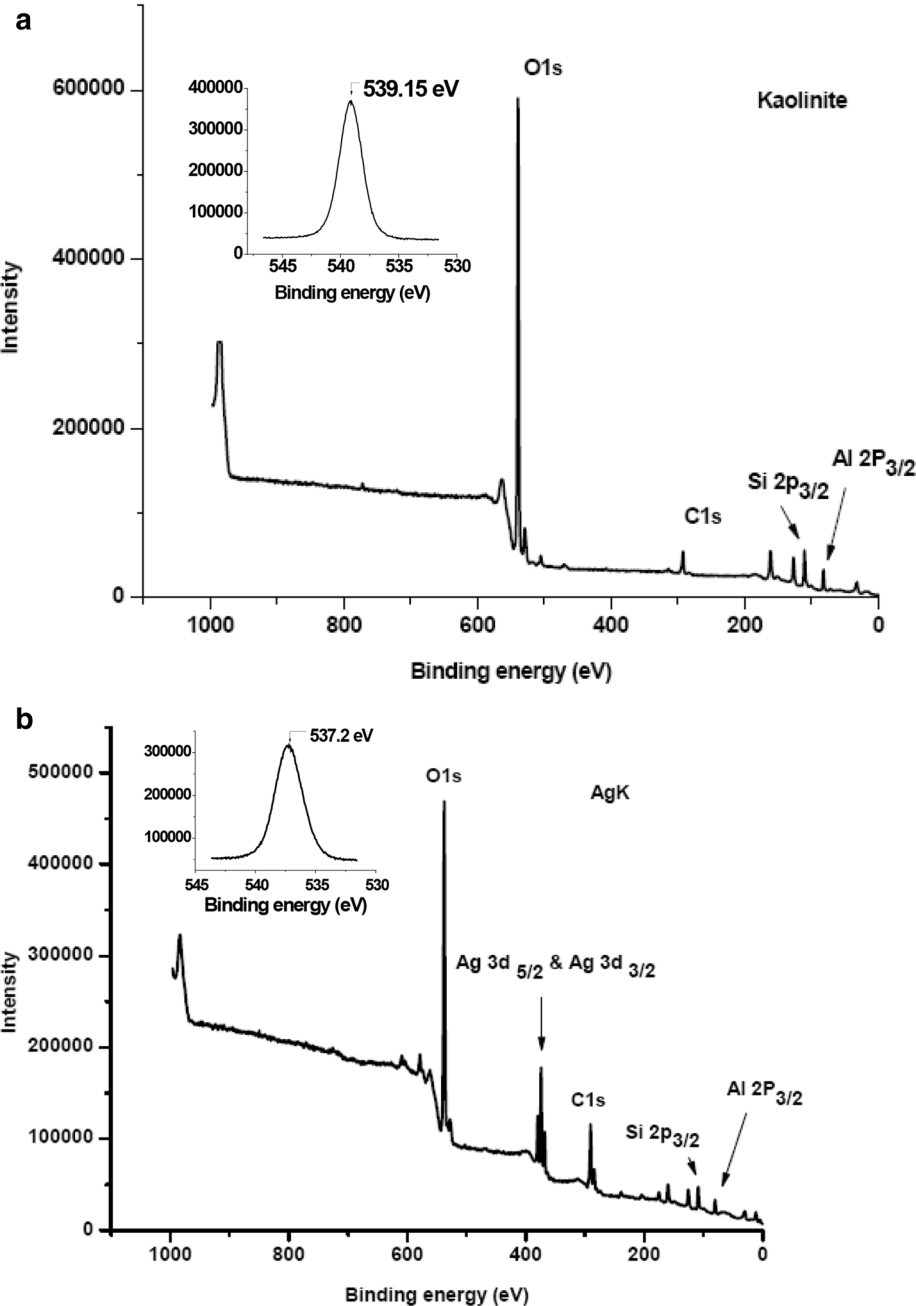
X-ray photoelectron survey spectra of **a** kaolinite and **b** AgK specimen

**Fig. 9:**
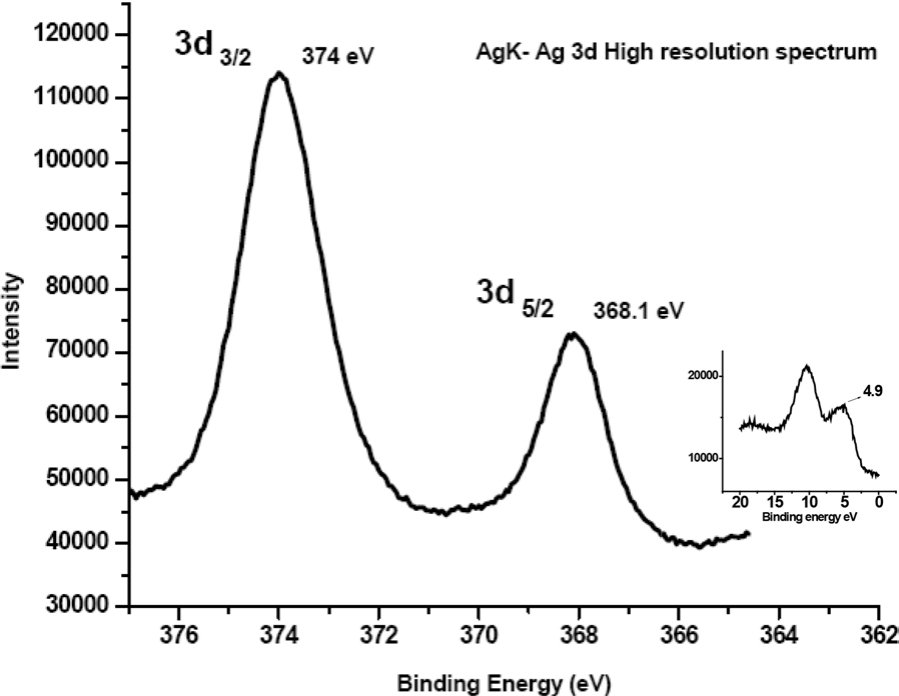
High resolution X-ray photo electron spectrum of silver (Ag) 3d and 4d (*insert*) of silver oxides obtained from AgK specimen

Figures 8a, b depict the photon emission survey spectra of kaolinite and AgK specimens. The XPS peak doublets at binding energies of 368.1 eV (electron volts) and 374 eV result from silver’s d-subshell spin orbital splitting 3d_5/2_ and 3d_3/2_ respectively (Wagner et al. 1979). The high resolution spectrum of silver’s d-shell spin orbital splitting is presented in Fig. 9. The FWHM (Full Width Half Maximum) of 3d_5/2_ peak corresponds to 1.45 eV. Hoflund and Hazos (2000), Bielmann et al. (2002) Weaver and Hoflund (1994) reported that the FWHM values of 3d_5/2_ peaks of Ag, AgO and Ag_2_O correspond to 0.57 eV, 1.57 eV and 1.5 eV respectively. According to available studies (Weaver and Hoflund 1994; Hoflund and Hazos 2000; Bielmann et al. 2002; Chiu et al. 2003; Al-Kuhaili 2007; Gao et al. 2004**)**, the relatively high FWHM of 3d_5/2_ peak (1.45 eV) in Fig. 8 suggests the existence of AgO and Ag_2_O in AgK specimen. As the photoemission spectra is a surface phenomenon (approximate penetration depth is 1–5 nm, Seyama et al. 2006; Vempati et al. 1996) the silver oxides in AgK specimen occur as surface coatings. The O1^s^ peak identified at 537.2 eV and 539.15 eV in silver–kaolinite and kaolinite respectively. The 4d peak of silver at 4.9 eV attribute to native silver oxides (Fig. 9; Vincent 2005).

### Electron probe micro analysis (EPMA)

**Fig. 10:**
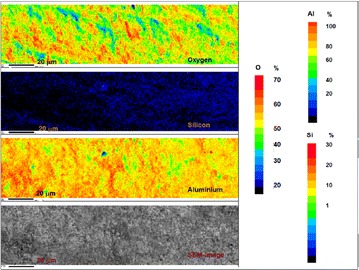
Wavelength dispersion elemental map of aluminium (*Al*), silicon (*Si*) and oxygen (*O*) obtained from kaolinite specimen

**Fig. 11:**
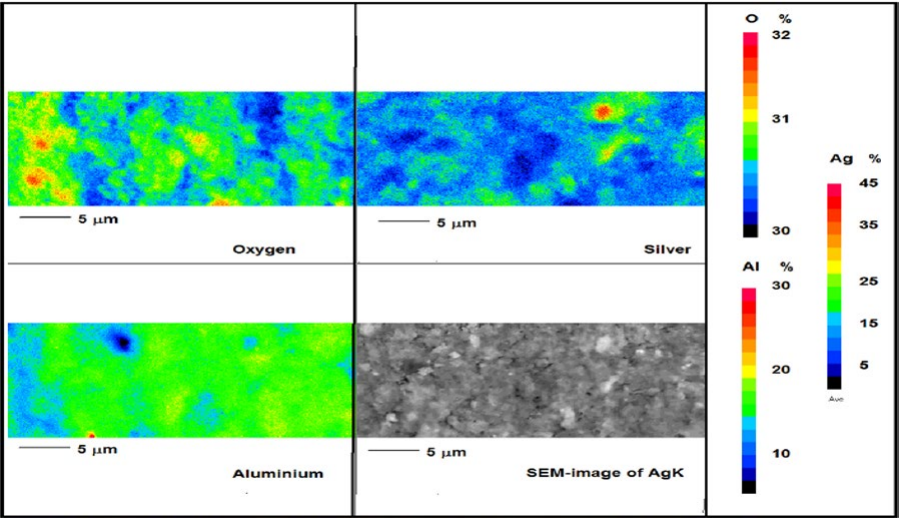
Wavelength dispersion elemental map of oxygen (*O*), aluminium (*Al*) and silver (*Ag*) obtained from AgK specimen

**Fig. 12:**
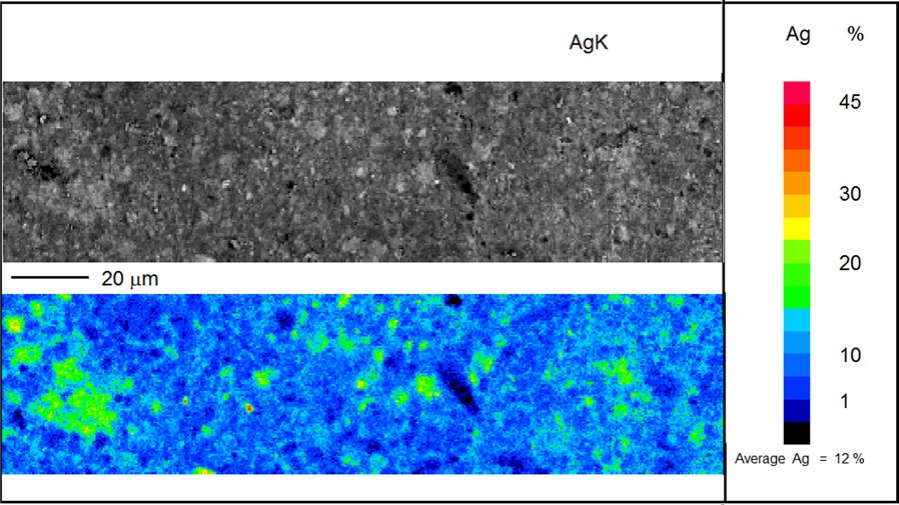
Wavelength dispersion elemental map of silver (*Ag*) obtained from AgK specimen

The wavelength dispersion X-ray elemental maps of kaolinite and AgK specimens along with backscattered electron images are presented in Figs. 10, 11 and 12. Figure 10 illustrates the distribution of silicon, aluminium and oxygen atoms on the kaolinite surface. Figure 11 illustrates the elemental map image of Si, Al, O and Ag distribution in AgK specimen. Figure 12 presents the elemental map of silver obtained from AgK along with scanning electron microscope (SEM) image of AgK which shows the distribution of silver on the surface of the AgK specimen.

### Thermal decomposition reaction of AgNO3

The weight loss calculations, XRD, XPS and EPMA analysis with kaolinite–silver nitrate mix/AgK/Kaolinite specimen suggest that the following speculative reaction for formation of uniform AgO/Ag_2_O coatings on surface of the AgK specimen:

5 $$2SiO_{2} \cdot Al_{2} O_{3} \cdot 2H_{2} O\, +\, 3AgNO_{3} \to 2SiO_{2} \cdot Al_{2} O_{3} \cdot AgO \cdot Ag_{2} O\, +\, 2NO_{2}\, +\, NO \,+\, 2H_{2} O \,+ \,O_{2}$$

The water molecules in Eq. (5) arise from dehydroxylation of kaolinite.

## Conclusions

The weight balance and thermal analysis showed that the silver retention on kaolinite surface is driven by salt-catalyzed dehydroxylation phenomena. The XRD pattern of AgK specimen indicated that the silver present in AgK specimen does not occur as Ag metal or AgNO_3_ molecule. The relatively high FWHM (Full Width Half Maximum) observed in the X-ray photon emission survey spectrum of AgK specimen suggested the existence of more than one silver oxide–AgO, and Ag_2_O in the specimen. As the photoemission is a surface phenomenon (approximate depth of penetration is 1–5 nm), it is inferred that the silver oxides in AgK apparently occur on kaolinite surface as silver oxide coatings. The electron probe micro analysis (EPMA) showed uniform distribution of silver on the surface of AgK specimen. The mass-balance calculations, XRD analysis, X-ray photon emission survey spectrum and EPMA tests with kaolinite–silver nitrate mix/AgK/Kaolinite specimen aided the formulation of chemical reaction for occurrence of uniform coatings of AgO/Ag_2_O on kaolinite surface of the AgK specimen. And also the AgK specimen would function as an additive to bentonite to improve its iodide retention capacity.
